# Vascular Progenitor Cells: From Cancer to Tissue Repair

**DOI:** 10.3390/jcm12062399

**Published:** 2023-03-20

**Authors:** Serena Barachini, Sandra Ghelardoni, Rosalinda Madonna

**Affiliations:** 1 Laboratory for Cell Therapy, Department of Clinical and Experimental Medicine, University of Pisa, 56126 Pisa, Italy; 2 Laboratory of Biochemistry, Department of Pathology, University of Pisa, 56126 Pisa, Italy; 3 Department of Pathology, Cardiology Division, University of Pisa, 56126 Pisa, Italy

**Keywords:** vascular progenitor cells, angiogenesis, cancer, metastasis

## Abstract

Vascular progenitor cells are activated to repair and form a neointima following vascular damage such as hypertension, atherosclerosis, diabetes, trauma, hypoxia, primary cancerous lesions and metastases as well as catheter interventions. They play a key role not only in the resolution of the vascular lesion but also in the adult neovascularization and angiogenesis sprouting (i.e., the growth of new capillaries from pre-existing ones), often associated with carcinogenesis, favoring the formation of metastases, survival and progression of tumors. In this review, we discuss the biology, cellular plasticity and pathophysiology of different vascular progenitor cells, including their origins (sources), stimuli and activated pathways that induce differentiation, isolation and characterization. We focus on their role in tumor-induced vascular injury and discuss their implications in promoting tumor angiogenesis during cancer proliferation and migration.

## 1. Introduction

When a vessel wall is injured, an intricate reconstitution process is triggered to repair and form a neointima. In this process, a leading role is played by vascular progenitor cells. Pericytes, mesenchymal stem cells, endothelial (EPCs) and smooth muscle progenitor cells (SMPCs) are a type of undifferentiated cells that are activated in the regeneration and replenishment upon vascular injury [1]. The organization of the vascular wall is well structured and consists of three different main functional layers: the intima, a single layer of endothelial cells, which provides an interface between blood and smooth muscle and contains vascular stem cells which are CD34 and c-kit-positive; the media, including smooth muscle cells, collagen and elastin; and the adventitia composed of undifferentiated dendritic cells, connective tissue, vasa vasorum and other cells including fibroblasts, pericytes, and cells CD34, Sca-1, c-kit, NG2 and GII1-positive. This well-organized vascular structure can deal with injuries that are often caused by several acute and chronic diseases including hypertension [2], atherosclerosis [3], diabetes [4,5], trauma [6], occlusions [7], hypoxia [8], primary cancerous lesions, and metastases [9] as well as catheter interventions [10,11].

To resolve the vascular damage, the progenitor cells are mobilized in the injured area and then they begin to differentiate and form a new vascular wall, with the aim of reorganizing themself into a new intima layer, the neointima [1,12]. The process is affected and regulated by the microenvironment, which maintains the dynamic equilibrium around the vessels: nerve fibers, adipose and immune tissues with the release of cytokines and vasoactive factors contribute directly or indirectly to the proliferation and remodeling of the injured vessel wall [13]. Vascular progenitors have a fundamental function not only in resolving the vessel lesion but also in adult neovascularization and in angiogenesis sprouting [12,14], which are often associated with cancerogenesis, and favor the formation of the metastases, survival and progression of tumors. Vasculature is important during tumorigenesis, facilitating the pool of nutrients and oxygen and the discarding of metabolic wastes [15]. Moreover, the formation of new capillaries is an essential driver of tumor growth. The sprouting of angiogenesis produces leaky and twisted vessels; this helps cancer cells relocate to other tissues and organs to produce metastases; on the other hand, it can lead to ineffective drug delivery within the tumor microenvironment [9,15]. 

Here, we elucidate the different vascular progenitor cells, including their origins (sources), stimuli and activated pathways that induce their differentiation, isolation and characterization. We focus on their role in tumor-induced vascular damage, while referring the reader to more specific reviews on intimal regeneration following vascular damage induced by other types of triggers (i.e., ischemia and reperfusion, sepsis, catheterization, etc.). Since the microenvironment influences vascular progenitor cell differentiation and their interactions, we discuss here their implication in promoting tumor angiogenesis during cancer proliferation and migration. 

## 2. Vascular Progenitor Cells

To date, in addition to circulating progenitors, different types of resident vascular stem/progenitor cells (VSCs) have been characterized and their role in vascular repair and the development of the disease has been evaluated. VSCs are defined as “cells that reside within the blood vessel wall and can differentiate into all of the cell types that make up a functional blood vessel” [16]. In particular, VSCs can differentiate into endothelial cells (ECs), pericytes in capillaries, smooth muscle and adventitia cells in larger vessels. Adult VSCs, quiescent in their niches, can be mobilized in response to injury or inflammation, to form new vessels during vascular repair. Several processes underlie vascular repair, and angiogenesis is the best known process. Angiogenesis is characterized by the sprouting of new capillaries from postcapillary venules [17] by in situ proliferation and the migration of pre-existing ECs. During embryonic development, the de novo formation of the blood vessel is defined as vasculogenesis, while in the adult it is postnatal vasculogenesis [18]. Moreover, arteriogenesis is the maturation and formation of larger diameter arteries from pre-existing capillaries or collateral arteries [19]. Finally, neovascularization includes all processes: angiogenesis, arteriogenesis and vasculogenesis [20].

Several studies have demonstrated that different subtypes of VSCs isolated from different tissues, such as EPCs, SMPCs and mesangiogenic progenitor cells (MPCs), can differentiate into various vascular cells to form the vascular wall in the course of vascular repair [21,22]. Three distinct layers form the vascular wall of arteries and veins: *tunica intima*, a single layer of ECs; *tunica media* composed of smooth muscle and elastic fibers; and *tunica adventitia*. In recent years, studies have shown that not only could the *adventitia* represent a complex depot for VSCs involved in endothelium repair [23], but also the *intima* is composed of VSCs that have an important role in the onset and progression of vascular remodeling diseases. VSCs could be the in situ equivalent of bone marrow (BM), MSCs or as perivascular stromal cells due to their perivascular location and multilineage potential [24]. Thus, the finding of VSCs suggests the presence of a vessel wall niche in which multipotent resident progenitor cells are capable under appropriate stimuli of regenerating small and large vessels [25]. 

In addition, VSCs also play a role in the angiogenesis involved in tumors. Several distinct biological processes, orchestrated by a series of secreted factors and signaling pathways also involving non-endothelial cells, progenitors or tumor stem cells, give rise to tumor vascularization [26]. Therefore, elucidating the biology of these VSCs is crucial to offer new potential therapeutic targets with relevant therapeutic implications in vascular diseases and regenerative medicine. In this regard, single-cell sequencing and cell lineage tracing could suggest the understanding of the origin and specific role of distinct VSC types.

### 2.1. Endothelial Progenitor Cells

Since their first identification in 1997 [27], EPCs have been extensively studied for their potential as endothelial lineage progenitors, having the ability to proliferate and differentiate into mature ECs in vitro, and to generate new vessels in vivo [28]. There are several studies regarding the biology of EPCs, the origin of surface markers and the hierarchy of differentiation, but there have been considerable controversies, especially in the different isolation method used and in the EPC origin. Table 1 reports the EPC markers in clinical trials. 

The adult hematopoietic system is a very heterogeneous and dynamic process. The existence of the hemangioblast, capable of differentiating into endothelial and hematopoietic cells, was shown two decades ago by Asahara and colleagues, who successfully isolated EPCs from human peripheral blood [29,30]. Since then, further studies support not only the existence of EPCs and their role in the formation of a new vessel [25], but the existence of a hemogenic endothelium (HE). HE has a hemogenic capacity and displays an Flk1+ (fetal liver Kinase 1) c-Kit+CD45−side population phenotype representing a heterogenous source of various types of progenitor cells generated not only during embryogenesis, but also in the BM of late fetus/young adults [25].

The heterogeneity of HE suggests that HE cells are already “primed” to become specific types of stem or progenitor cells when still undergoing the endothelial-to-hematopoietic transition (EHT), a process involving the transcription factor Runx1. HE cells undergo an EHT giving rise to hematopoietic stem cells (HSCs) with intermediate stages, such as pre-HE, revealed by scRNA-seq studies showing the differentiation trajectory of EHT [31,32]. Subsequently, hemogenic angioblasts are thought to migrate within the fetal liver and fetal BM as these tissues develop. Recently, Zhao et al. [33], using photoconvertible labeling, time-lapse imaging and a single-cell RNA-sequencing analysis in a zebrafish model, found that HE and ECs arise from a common flk1+ precursor, which they refer to as hemogenic angioblasts, and their distinct fate is regulated by ETS transcription factors Etv2. Interestingly, Etv2 has been found to be involved in tumor angiogenesis [34]. 

EPCs were originally shown as circulating cells that have the potential to differentiate into mature ECs, being involved in postnatal vasculogenesis. Most of the scientific studies, regarding the biology of EPCs, used CD34+ or CD133+ cells in human peripheral blood mononuclear cells and found their commitment into the endothelial lineage cell in vitro and their integration into EC in the formation of a new blood vessel in vivo [35], supporting the evidence that cells derived from CD34+ are incorporated into the human vasculature. However, EPCs have been shown to be actually a mixture of several cell types, such as early and late EPC, explaining some contrasting results due to the inconsistency of culture condition standardization [14,36].

EPCs have been isolated from different sources: bone marrow, spleen, umbilical cord blood, adipose tissue, placenta. Ingram et al. [37] revealed intima-derived EPCs expressing CD31, von Willebrand factor (vWF), CD146, Flk-1, CD144 and CD105, suggesting a hierarchical organization of different EPC subpopulations. Subsequent data of Bearzi et al. [38] demonstrated that all three mural layers of human coronary arteries include small groups of clonogenic VSCs, expressing c-kit+ VEGFR2+ and CD45+ that are able to differentiate between ECs and SMCs. 

The early theory about the use of circulating EPCs for vascular repair has been challenged by later studies indicating that CD34+ cells derived from BM were more likely to differentiate into inflammatory cells and not EPCs. Instead, high-proliferative and pro-angiogenic EPCs within the vessel wall have been found to have a similar clonogenic capacity to repair endothelium. Further research has suggested that CD34+ cells may be a tissue-resident source for EPCs. EPCs are promising for cardiovascular therapy, but clinical research on EPC-captured stents has shown limited efficacy. However, CD34+ cell transplantation in myocardial tissue has been shown to be safe and efficient in patients with heart disease [36].

Recently, embryonic-like stem cells (VSELs) [39] have been identified as very small cells (5–7 μm) with large euchromatin-containing nuclei and cytoplasm enriched in mitochondria expressing pluripotent octamer-binding transcription factor 4 (OCT-4) [40]. VSELs have been proposed to originate from germline-related cells and play a role as a backup population for tissue-committed monopotent stem cells. VSELs are deposited in developing organs during embryogenesis as highly quiescent cells but are mobilized into circulation during stress stimuli. It has been speculated that VSELs are at the top of the stem cell hierarchy in BM, lacking the CD45 marker on their surface, and give rise to HSCs, EPCs and MSCs. VSEL specification in HSCs and EPCs may involve a presumed hemangioblast as an intermediate cell.

EPC has been shown as a therapeutic option to improve tissue function by enhancing angiogenesis, as well as neurogenesis in ischemic stroke and brain damage models [36]. In addition, EPC and MSC implanted together contribute to vessel formation in the ischemic muscle more effectively than EPC alone [41,42].

Until now, results are often conflicting, do not cover all the possible functions and applications of EPCs due to different isolation protocols and lack both a precise surface marker and a functional assay. At present, there is a need for a new classification of EPC according to their origin, phenotype and function, while also redefining EPCs with state-of-the-art multiomics technology. 

### 2.2. Smooth Muscle Progenitor Cells

In new vessels, smooth muscle cells stabilize mechanically the vascular wall and help in regulating vascular tone and blood flow. A long-held view was that SMCs consist of heterogeneous populations with remarkable plasticity, and exhibit either a proliferative and migratory phenotype with the presence of stem cell markers, or a quiescent, contractile and mature phenotype [43]. Table 2 reports SMC markers in clinical trials. As reported in several investigations, when blood vessels are injured, VSCs can move to the intima, starting cell proliferation, by interchanging from a contractile phenotype to a secretory one. Conversely, another study showed that VSCs are involved in vascular remodeling without phenotypic switching [44]. Abnormal phenotype switching is a feature in the etiology of several different diseases.

There are different views regarding the origin of SMCs. Some suggest that they are heterogeneous, while others propose that they originate from multipotent vascular stem cells and differentiate into specific subpopulations with distinct functions. Early studies in mice indicated a predominant role for BM as a potential source of vascular progenitors [45]. Subsequent studies, however, failed to reproduce these data [46]. Other reports have identified vascular SMPCs in microvascular pericyte, adventitial MSC and vascular ECs, suitable for the endothelial-to-mesenchymal transformation (EndMT) [25]. Furthermore, recent data showed JAG1-induced Notch signaling is critical for the regulation of EPC differentiation and proliferation and promotes MSC differentiation into SMCs [47]. 

Recent investigations showed the *adventitia* in the vessel as a complex layer of wall consisting of different types of cells, including resident progenitor cells, located in a specialized niche at the *media–adventitia* border. More than a decade ago [48], Sca1+ cells in the adventitial layer of the vessel wall were proposed as stem cells for SMCs although it was not proven to have the in vivo function in vascular injury and vessel remodeling, due to technical limitations at that time.

Later, Kramann et al. [49], based on a lineage tracing study, demonstrated that Gli1+ MSC-like cells are adventitial progenitor cells; CD34, Sca-1 and PDGFR-β (platelet-derived growth factor receptor β)-positive; collaborating to neointima production and to restore after an acute damage to the femoral artery. Adventitial progenitors express Sca1 and CD34 and show a multipotent phenotype suitable to differentiate in vivo into adult SMCs, contributing to both intimal and adventitial remodeling [50]. The pluripotency-associated transcription factor, Klf4, is required for the preservation of the adventitial Sca1 progenitor cell phenotype. However, the origin of adventitial Sca1 progenitor cells remains unclear. Recently, Tang et al. [51], using single-cell RNA sequencing and genetic cell lineage tracing, proved that adventitial Sca1+ VSCs help in the formation of SMPCs and play a crucial role in vessel repair, identifying a potential therapeutic target for the treatment of cardiovascular diseases.

The *adventitia* may be a compartment that provides an appropriate microenvironment for VSCs [52], where Sca1+ progenitor cells and other adventitial stem cell populations are a backup system operating in conditions of loss or insufficiency of SMCs. This is also the site for the restricted domain of sonic hedgehog (Shh) signaling, which may provide a pivotal contribution in preserving vascular SMC progenitor cells resident in the artery wall [53,54]. In addition, SMCs, pericytes and ECs may have a common progenitor, namely, Flk1-positive embryonic cells [55] where FLK1 maintains the Sca-1+ progenitor cell phenotype.

VSCs in the *adventitia* can migrate into the *media* and differentiate into vascular SMCs facilitated by the disruption of the elastic lamellae to support their migration. A few expanding cells were found to be the origin of oligoclonal SMCs in injury-induced neointimal lesions and atherosclerotic plaques, as demonstrated by a recent paper. These cells could either be progenitors of quiescent SMCs or have the ability to dedifferentiate into progenitors [56]. SMCs in lesions comprise all the stages of differentiation, thus exhibiting a variety of phenotypes [52]. The elucidation of molecular pathways driving the plasticity, heterogeneity and differentiation of SMPCs may lead to the finding of new and more peculiar targets for disease prevention and treatment.

### 2.3. Mesangiogenic Progenitor Cells

The perivascular localization of mesenchymal precursors may justify their presence in a wide range of tissues and organs and suggest an angiogenic potential. MSCs are pluripotent, self-renewing, spindle-shaped cells isolated from adult tissues such as BM, adipose tissue and dental pulp and from perinatal tissues such as umbilical cord blood, placenta, amniotic fluid and umbilical cord Wharton’s jelly [57,58,59,60]. The features of MSC from various organs are different in phenotypes, differentiative potential and result from the influence of a local environment.

MSC derived from adventitial reticular cells express CD271 and CD146 markers and are located in the subendothelial layer of the sinusoids [61]. Moreover, another different population expressing CD271 but not CD146, located in the endostal niche, is able to generate MSCs [62]. In addition, two intra-vessel wall compartments, the *adventitia* and *subendothelium*, have been suggested as possible sites for MSC progenitors. However, a defined characterization of MSC and standardized protocols for their isolation and expansion are still lacking. BM-derived MSCs can differentiate into ECs and SMCs in vitro via modulation of growth factors. Non-medullary progenitor cells with pro-angiogenic capacities have been reported as CD34-positive/CD31-negative cells, expressing pericyte/MSC markers together with Sox2 and located in human saphenous veins [63]. It is now increasingly recognized that MSCs typically do not engraft after transplantation and have limited capabilities to trans-differentiate in vivo, but they exhibit their therapeutic effect in a paracrine manner through the secretion of bioactive factors (such as microRNA, transfer RNA, long non-coding RNA, growth factors, proteins and lipids) collectively referred to as the secretome [64]. Moreover, there are conflicting data regarding their angiogenic potential due to the heterogeneity of the primary MSC cultures utilized to generate endothelial progenitors.

We recently isolated a new population of progenitors of mesengenic lineage progenitor cells from human BM with angiogenic potential, named mesangiogenic progenitor cells (MPCs), which are tissue-specific [65,66,67]. We hypothesized that the presence of the MPCs could be responsible for conflicting data regarding the angiogenic potential of MSC cultures. By replacing fetal bovine serum with pooled human AB serum [68] in the culture medium of human BM-mononuclear cells, we obtained a Ki-67-negative, adherent cell population with long telomeres, condensed chromatin and podosomal structures [69]. MPCs are quiescent, fried egg-shaped cells, positive for Nestin, CD31 and CD105 but negative for the expression of CD73, CD90, CD166 and CD271 and other typical markers for a mesenchymal phenotype such as MSCA-1. Table 3 reports MPC markers in clinical trials. Interestingly, MPCs were positive for the pluripotency-associated transcription factors Oct-4, NANOG and to a lesser extent c-MYC, but lacked typical MSC markers, such as RUNX2 and Sox2 [70]. In addition, this cell population, when human AB serum was replaced with fetal bovine serum, rapidly produced MSCs in a multistep hierarchical pattern with an intermediate cell population, *early* MSC, whose fate was regulated by a non-canonical Wnt5/Calmodulin pathway [71]. *Late* MSC, unlike MPC, did not have strong expression of aldehyde dehydrogenase (ALDH), a stem/progenitor cell marker expressed also by HSC and EPC [72], and could not be induced to differentiate into MPCs. Similarly, other research groups [73] isolated the cardiac-derived ALDH+ cell population in mouse hearts, whose expression decreased with cell passage, suggesting a similar loss of ALDH expression and cell potency over time. In addition, two populations of EPCs, CD31+, CD105+ and CD45- have been isolated from human umbilical cord blood, with low or high ALDH activity (Alde-Low and Alde-High) [74]. Alde-Low EPCs had a highly proliferative and migratory ability and were more responsive to hypoxia compared with Alde-High EPCs.

The angiogenic potential of MPC was demonstrated by the standard angiogenesis assay in which MPCs formed capillary-like structures after multiple steps of differentiation [75]. Indeed, MPCs can begin sprouting when directly seeded in Matrigel 3D cultures, but they are unable to efficiently form tube-like structures without a differentiation step, suggesting that MPCs are a stem progenitor with angiogenic potential. MPCs have been shown to derive from a single-BM-cell population named *Pop#8* [76]. This cell population has been sorted from adult human BM as CD45^low^CD31^bright^CD64^bright^CD14^neg^ and showed similarities to monocytoid progenitors. Gene expression data suggested the in vivo involvement of *Pop#8* in maintaining the hematopoietic stem cell niche. Moreover, the high expression of CD31 and Nestin in MPCs suggests that these cells may represent a primitive progenitor for endothelial lineages. Nestin was originally reported as a 176 kDa class VI intermediate filament protein in neural stem cells of the embryonic and adult brain, and later in ECs [77]. We also demonstrated that Nestin expression in benign human BM biopsies was detected in the ECs of small arteries and endosteal arterioles, and also in very small vessels, named NESTIN+ capillary-like tubes, suggesting Nestin is an associated marker of neovascularization [78]. 

A subset of progenitors with mesengenic potential has been isolated among BM-Nestin+ populations, as reported by Mendez-Ferrer et al. [79]. These cells express high levels of CXCL-12 and Ang-1, but they lack endothelial markers such as CD31, vascular endothelial cadherin or CD34 and limited data are available regarding their angiogenic potential [79]. In 2013, Kunisaki et al. [80] identified specialized perivascular populations using Nestin-GFP mice. They found that Nestin is highly expressed in quiescent cells near arterioles (NES-peri), while perivascular reticular cells closely related to sinusoids have a higher proliferation rate but a dim expression of Nestin (NES-retic). 

Recently, by combining single-cell and spatially resolved transcriptomics [81], Cxcl12-Abundant-Reticular (CAR) cell subsets have been isolated in BM niches, i.e., Adipo-CARs localizing in the sinusoidal endothelium and Osteo-CARs localizing in the arteriolar endothelium. These cells *behave* as “professional cytokine-producing cells” and constitute perivascular micro-niches involved in the maintenance and differentiation of HSC. 

Finally, MPCs are believed to be the ancestors of MSCs, and although their in situ localization has not been fully understood, it is probable that they reside in the *tunica intima* of BM vessels, in contact with CAR cells or the NES-peri population.

### 2.4. Pericytes

Pericytes coat microvascular capillaries, where they determine the formation, maturation, maintenance, stabilization and remodeling of the vascular system. They come into direct contact with underlying ECs, share some properties with MSCs and could trans-differentiate into myofibroblasts, SMCs and adipocytes, therefore modulating the vascular net and flow [82]. 

Classical markers that identify pericytes are CD13, CD146, α-SMA (smooth muscle α-actin), PDGFR-β, NG-2 (Neuron-glial antigen 2) and desmin, while other new markers are RGS5 (regulator of G protein signaling 5), DLK-1 (delta-like homolog 1) and Endosialin (CD248 or TEM1), but none of them can definitively define them due to their overlap with the other markers of other adjacent cells. Table 4 reports pericyte markers in clinical trials. Pericytes not only regulate and promote angiogenesis through extracellular matrix (ECM) and ECM-associated factors, but they also secrete bioactive molecules such as cytokines, angiogenic and growth factors [83]. These bioactive molecules promote local proliferation and differentiation of MSC. 

Pericytes play a pivotal role in both the vessel sprouting and stabilization phases of angiogenesis; the latter is initiated by the release of matrix metalloproteases from ECs and pericytes, allowing pericyte detachment and a transition from their quiescent phenotype to the actively proliferating one. Several angiogenic factors induce the migration of ECs and one single EC “tip cell” and recruit other ECs through the VEGF (vascular endothelial growth factor) gradient. The other ECs are called “stalk cells” and form the growing lumen. Vessel maturation is achieved via the secretion of TGF-β (transforming growth factor-β) and ANG1 (angiopoietin1) by ECs and pericytes [84]. 

The PDGF family has four members (A, B, C, D), and they activate two receptor tyrosine kinases (PDGFR-α and PDGFR-β) to induce downstream signaling pathways. PDGF-β dimer is a key regulator of vascular pericytes and is required for their recruitment to nascent blood vessels [47]. PDGF-C is also important in regulating VSCs and enhances their proliferation, migration and differentiation into ECs. PDGF receptors have been shown to have important effects on VSCs, and their overexpression increases the proliferation and migration of VSCs via ERK and PI3K/AKT activation. In addition to the mediating effects of PDGF, PDGF receptors can also mediate those of VEGF-A, promoting the migration and proliferation of MSCs via PDGFR-α and PDGFR-β [47].

The VEGF signaling pathway plays a critical role in vasculogenesis and angiogenesis, starting with the expression of VEGF receptors (VEGFRs) on primitive ECs. VEGF-A is the prototypical member of the VEGF family and is released in response to hypoxia or hypoperfusion of a tissue. VEGFR2 is the most important signaling receptor in vasculogenesis and angiogenesis, with high intracellular tyrosine kinase activity when activated. The knockout of VEGFR2 or VEGF-A results in early embryonic lethality due to the lack of organized vasculature [85]. Intracellularly, VEGFR2 activation propagates via a number of downstream signaling pathways, such as ERK signaling, inducing endothelial proliferation and differentiation and leading to the expression of cell adhesion molecules such as VE-cadherin. The control of the VEGFR expression is maintained by exogenous signaling such as the fibroblast growth factor (FGF) and TGF–β and negative feedback loops within the cell. Perivascular cells also express VEGF, primarily VEGF-A, which stabilizes newly formed vessels but does not promote endothelial cell migration. Pericytes have VEGFR1 on their cell surface, which sequesters VEGF from VEGFR2 on endothelial cells, preventing the initiation of angiogenesis in mature, quiescent vessels [85].

New investigations have reported that pericytes derive from human pluripotent stem cells, developing into MSCs and lastly differentiating into immature pericytes and SMCs. Immature pericytes can vary into type I and II pericytes, which propagate towards several tissues and organs where they carry out their functions [86].

Herrmann et al. [87] compared and characterized pericytes isolated from BM and adipose tissue as CD45-CD34-CD146+ cells. Their results showed that BM-derived cells demonstrated triadic differentiation potential, while adipocyte-derived cells had poor chondrogenic differentiation. This suggests that the microenvironment of the organ of origin may impact cell processes.

Recent studies using lineage tracing experiments have shown that the cell fate plasticity of endogenous pericytes in vivo is unclear. The transcription factor *Tbx18* has been found to selectively mark pericytes and SMCs in multiple organs of adult mice [88], indicating that pericytes do not behave as tissue-resident multipotent progenitors. These findings highlight that the plasticity observed in vitro or following transplantation in vivo may be a consequence of the artificial cell culture environment. 

## 3. Vascular Progenitor Cells in Cancers

**Figure 1:**
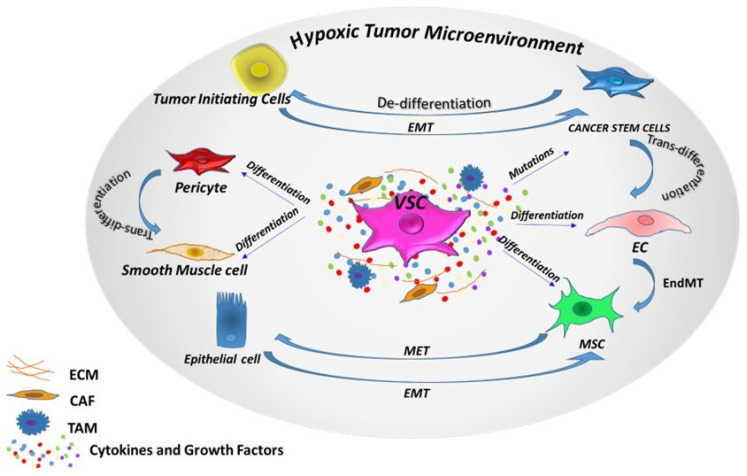
Cellular plasticity. Phenotypic plasticity can enable stem cells and non-stem cells to shift among one another via different programs, depending on intrinsic (epigenetic) and extrinsic (tumor microenvironment) factors. VSC: vascular stem/progenitor cells; EC: endothelial cells; MSC: mesenchymal stem cells; TAM: tumor-associated macrophages; CAF: cancer-associated fibroblast; EMT: epithelial-mesenchymal transition; MET: mesenchymal-epithelial transition; EndMT: endothelial-mesenchymal transition.

Recent findings in oncology have considered the extensive heterogeneity of tumors, with the “cancer stem cells plasticity” model being responsible for maintaining this heterogeneity, fueling tumor growth and therapy resistance. In this model, cancer stem cells shift between stem and differentiated states, induced by both intrinsic factors such as genetic mutations and/or epigenetic modifications and extrinsic factors from the tumor microenvironment (TME) (Figure 1). Switches between these states can occur via different programs: de/transdifferentiation, asymmetric/symmetric division, quiescence/proliferation, epithelial-mesenchimal transition (EMT)/mesenchymal-epithelial transition (MET) and drug sensitivity/resistance [89]. After formation, solid tumors continue to interact with various stromal cells, including local and infiltrating fibroblasts, macrophages, MSCs, ECs, pericytes and secreted factors within the TME, promoting tumorigenesis and inducing resistance to therapy [15]. In fact, TME plays a critical role in modifying and manipulating different tumor niches, which in turn affect the behavior of cancer stem cells. TME signals, such as TGF-β, promote the chromatin configuration in non-cancer stem cells to activate genes such as ZEB1, one of the key EMT regulators essential for maintaining stemness plasticity in the cells. This process switches non-cancer stem cells toward a cancer stem cell phenotype [90]. A high level of TGF-β promotes EMT, a trans-differentiation program, to disseminate toward the distant sites (early metastasis), while low TGF-β induces MET to maintain self-renewal in the sites of invasion (late metastasis). Cancer stem cells exhibit different transcriptional and epigenetic signatures depending on the niche from which they derive, and engage in a complex array of signaling with both tumor cells such as cancer-associated fibroblasts (CAF), tumor-associated macrophages (TAM) and normal cells such as immune cells, differentiated cells, ECs. Ghajar et al. found that ECs use signaling pathways such as Notch, sonic hedgehog and nitric oxide to promote a stem-like phenotype in breast cancer cells, while mature blood vessels can induce dormancy in breast cancer cells through thrombospondin-1, an angiocrine tumor suppressor [91]. 

Both stem cells and cancer cells have the ability to self-renew and share signaling pathways commonly associated with stem cell replication such as Wnt, Bcl-2, Shh and Notch [92]. Single-cell sequencing studies have identified diverse clonal populations of cancer cells with stem cell-like profiles [93]. The mutagenesis in cancer cells contributes to their diversity, but it is also likely that this heterogeneity is the result of incomplete or aberrant cellular differentiation. The trans-differentiation of cancer cells along the endothelial cell lineage to support tumor angiogenesis represents a further avenue to cancer cell plasticity (Figure 1). Liu et al. showed, in a CD133-positive cancer stem subpopulation of triple-negative breast cancer, the capability to form tube-like structures [94]. Additionally, in renal cell carcinoma, the expression of CD133 and CD44 was associated with high cancer stem cells’ marker expression and angiogenic structures correlating with poor survival. These findings demonstrate that cancer stem cells can not only interconvert among their subpopulations but can also generate different kinds of non-cancer stem cells with a differentiated phenotype [90].

Furthermore, it has been hypothesized that VSCs may be recruited by tumor cells to participate in the formation of new blood vessels necessary for tumor growth. Understanding the role of various VSCs is crucial in delineating the complex and dynamic process of tumorigenesis, which involves cell–cell and cell–extracellular matrix interactions facilitating tumor cell growth, drug resistance and metastasis.

Blood vessel formation is essential for tumor growth, as it depends on a proper nutrient and oxygen supply, and VEGF is a master regulator of angiogenesis. The tumor vasculature is fundamentally different from normal vasculature [95], characterized by uncontrolled and disorganized blood vessel networks that are “leaky” and convoluted. This abnormal vasculature often leads to impaired blood flow, causing inadequate oxygen supply to tumor cells and resulting in microregional hypoxia [96]. As a consequence, resistance to both radiotherapy and chemotherapy is common. The interplay between pro- and anti-angiogenic factors modulates the rate of angiogenesis in different tissues, making the angiogenesis process a critical event for progression and metastasis [97]. Among these factors, VEGF is a major and potent pro-angiogenic factor in tumors, while PDGF and FGF are other significant pro-angiogenic factors related to VEGF. The maintenance of a balance between pro- and anti-angiogenesis factors is essential to prevent tumor overgrowth and induce a state of tumor dormancy [98]. However, perturbation of this equilibrium leads to increased angiogenesis and uncontrolled tumor overgrowth. VEGF contributes to tumor initiation by promoting cancer stem cell function through a complex autocrine and paracrine signaling pathway, as well as initiating tumorigenesis by contributing to EMT activation. The angiogenic switch, which is a part of multistage tumorigenesis, arises from an imbalance between pro- and inhibitors of angiogenesis activity and is regulated by stromal cells of the TME.

Furthermore, cancer-associated ECs have been found to promote tumorigenesis through immunosuppression, synthesis of growth factors and enhanced migratory behavior of cancer cells [99]. Matsumoto identified tumor-specific blood ECs in a mouse glioma model, that originated from cells expressing the receptor for colony stimulating factor 1, *Csf1r*, (a cytokine that controls macrophage biology), demonstrating that these cells formed tumor vasculature and their selective depletion reduced vascular branching and tumor growth [100]. In the tumor mass, MSCs and HSCs act as angiogenic stimulators by secreting VEGF-A, supporting tumor vascularization. On the other hand, EPCs are the primary agents of lumen formation in new angiogenic sprouts [101]. The paracrine crosstalk between tumor and stromal cells results in accelerating tumor progression.

Chen et al. [102] revealed that microvascular ECs are not only the target of pro-angiogenic factors, but also the cellular source of pro-angiogenic factors that involve epithelial proliferation, angiogenesis, stem cell maintenance and stromal remodeling in lung and colon-rectal cancer. High levels of VEGF produced by tumors mobilize BM-derived EPC to enter the peripheral circulation, and enhance the recruitment of these cells to tumor sites. Hypoxia in tumors and HIF-1α activation promote the secretion of SDF-1α and VEGF, which can stimulate EPC mobilization and recruitment. To date, both SDF-1α/CXCR4 and VEGFA/VEGFRs pathways are the principal mediators of BM-derived EPC mobilization during cancer development and represent potential targets for novel anti-vasculogenic therapies [103]. The EPCs, in turn, host the cancer site, where they are incorporated into new vessels, and produce other growth factors that instruct neighboring capillary ECs to proliferate. Thus, in the early stage of cancerogenesis, EPCs are in higher concentrations than in later stages. 

Currently, there are two opposing views on how EPCs repair damaged vessels and promote neovascularization: one suggests that they differentiate into ECs and integrate directly into injured vessels, while the other suggests that they release paracrine substances. EPCs secrete various cytokines, growth factors, lipids and ECM, providing nutritional and anti-apoptotic support for circulating resident EPCs and other cells such as ECs, cardiomyocytes, MSCs and neural stem cells. EPCs’ paracrine production can be categorized into three types: (1) secretion of cytokines such as VEGF, SDF-1, PDGF, G-CSF, growth factors and chemokines to promote angiogenesis and EC proliferation and migration; (2) participation in intercellular communication by secreting extracellular vesicles such as apoptotic bodies, microvesicles and exosomes; and (3) connecting to ECs via nanotubes which allow the transfer of molecules such as proteins, RNA and other substances between cells [104].

The unfavorable microenvironment, characterized by hypoxia and acidosis, may affect the efficacy of chemotherapy and radiotherapy, and may interfere with the stimulation of cytotoxic activity during immunotherapy. Another important element to consider is the intrinsic plasticity of VSCs in response to the selective pressure exerted by the therapy and the microenvironment itself. While angiogenesis has long been considered to be promoted only by the stimulation of mature ECs, recent studies have shown the role played by the hypoxic tumoral ecosystem that modifies the secretome, proteome and metabolome of VSCs by reprogramming them into pro-tumorigenic phenotypes. This process may be a possible way through which VSCs and tumor plasticity are connected. Several studies have shown, in human liposarcoma and melanoma, the presence of alterations in specific tumor ECs: a heterogeneous population probably trans-differentiated from VSCs [105]. Our research group also identified specific MPCs in multiple myeloma (MM), demonstrating their involvement in both tumor growth and osteolysis [106]. These MPCs contributed to the formation of new blood vessels and reduced osteoblastogenesis. Interestingly, MM-derived MPCs exhibited greater angiogenic potential compared to patients with a non-hematological disease. It is possible that the behavior of MPCs is altered during the development and progression of MM due to the deregulation that malignant plasma cells exert on the stroma of the TME. Moreover, it is not uncommon for different progenitors to share or change markers, highlighting the mutual influence between various cell types and their coevolution in tumor development.

Pericytes also have multiple roles within the TME, including the coverage of ECs along the surface of the endothelium, basement membrane remodeling during tumorigenesis and new blood vessel formation. Although several clinical studies targeting the involvement of pericytes in angiogenesis have been performed, the results are unsatisfactory due to a heterogeneous and undetected population [15]. Therefore, powered clinical trials targeting the correct pericyte phenotype are needed to stop tumorigenesis. 

Several studies have shown that OCT-4-positive VSELs have been implicated in the initiation of cancers in various organs [107,108]. The uncontrolled expansion of these multipotent progenitors after insult leads to cancer initiation, which changes VSELs into cancer stem cells expressing CD 133, CD166.

**Figure 2:**
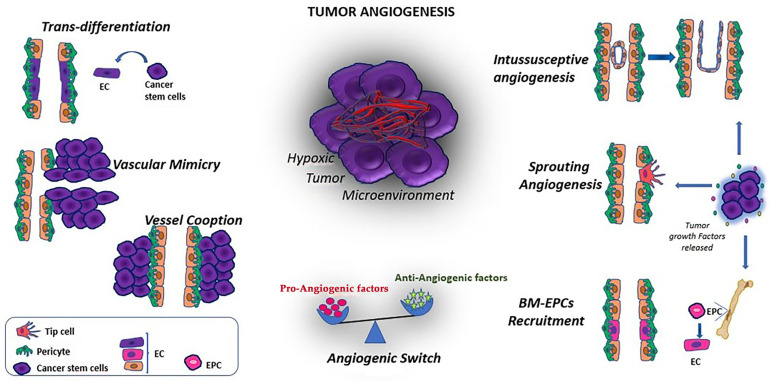
Mechanisms of tumor angiogenesis. Angiogenic and growth factors produced by tumor cells, hypoxia and acidosis in the tumor microenvironment promote the sequence of alternative mechanisms of angiogenesis. Trans-differentiation of cancer stem cells: proteins expressed in tumor cells induce trans-differentiation of cancer stem cells to endothelial cell-like phenotypes. Vascular mimicry: a matrix-embedded fluid-conducting meshwork formed by tumor cells. Vessel cooption: tumors gain blood supply by hijacking existing vasculature, and tumor cells migrate along the existing vessels. Sprouting angiogenesis: a process involving outgrowth of tip cells from the existing vasculature directed towards areas with a high abundance of pro-migratory growth factors released by the tumor. Intussusceptive angiogenesis: the splitting of the pre-existing vessel into two vessels. This process is characterized by the formation of ”transvascular pillars” without endothelial proliferation. BM-EPC recruitment: angiogenic and growth factors recruit, from BM, EPCs that differentiate into ECs. (EC: endothelial cell; BM: bone marrow; EPC: endothelial progenitor cell).

Additional tumor angiogenesis mechanisms involving cellular plasticity that can occur in tumors are vascular mimicry (VM), vessel cooption and intussusceptive angiogenesis [109] (Figure 2).

Vascular mimicry (VM) is an alternative blood supply system that is associated with invasion, metastasis, poor prognosis in cancer patients and resistance to anti-angiogenesis drugs [110]. It is a process in which tumor cells form tube-like structures to obtain nutrients without the involvement of endothelial cells, suggesting that malignant cells acquire an endothelial-like phenotype via trans-differentiation (Figure 2). VM has been identified in many cancers, including ovarian, breast, prostate, melanoma and glioma [111]. However, the presence of VM is not universally accepted, despite claims of its identification through strong positive staining for glycoproteins such as Periodic Acid Schiff (PAS) in animal and human cancers. One of the important molecules in VM formation is VE-cadherin (CDH5, CD144 commonly expressed by ECs), which is used as a biomarker for VM, other than ephrin type-A receptor 2 (EphA2). VEGF-A has been associated with VM formation via increasing the expression of metalloprotease, VE-cadherin, EphA2 and angiopoietin/Tie receptors. The ECM by itself can play a fundamental role in regulating VM. TAMs and CAFs appear to promote VM formation in several cancer types. In addition, MSCs have been demonstrated to migrate to the cancer microenvironment and play a role in supporting the establishment of the tumor vasculature, as well as suppressing immune responses, which can ultimately modulate the tumor’s response to anti-cancer therapy [112]. In addition, a recent study, using a single-cell transcriptome analysis, classified tumor endothelial cells into three subpopulations that suggested correspondence with tip-like, transitional and stalk-like cells suggesting a heterogeneity of ECs in tumors. The authors also demonstrated that anti-VEGF treatment reduced the number of endothelial tip-like cells in a tumor xenograft [113]. Finally, within the ECM tumor networks, recent data [114] showed that PAS+ tissues in human cutaneous melanoma stained positive for the pericyte marker α-smooth muscle actin. Here, pericytes were important players in VM, where tumor cells recruited them by PDGF-B signaling to support the formation of vascular-like networks and promote tumor sprouting. Therefore, novel anti-tumor neovascularization strategies need to target all alternative mechanisms, including VM, in addition to conventional angiogenesis. However, identifying VM in cancers remains controversial due to difficulties distinguishing VM tube-like structures from endothelial blood vessels in vivo. In vitro research, using 3D models with Matrigel, may also not always reflect in vivo VM, leading to inherent drawbacks. 

Vessel co-option, also known as vascular co-option, is a mechanism by which tumors obtain a blood supply by hijacking existing vasculature. Tumor cells migrate along the vessels within the surrounding host tissue without the need for angiogenic growth factors (Figure 2). This process has been observed in many malignancies, particularly in highly vascularized tissues such as the brain, lung and liver, where cancer cells can co-opt pre-existing blood vessels and capillaries [115]. Preclinical models have shown a switch from angiogenesis to vessel co-option during anti-angiogenic treatment such as anti-VEGF therapies [116]. During vessel co-option, ECs and pericytes play specific roles. Pericytes may support co-opted vessels by promoting EC survival through autocrine VEGF-A signaling [117].

Intussusceptive angiogenesis, related to the formation of new vasculature in which a pre-existing vessel divides in two (Figure 2), has been shown in various cancer types including melanoma, colorectal cancer, glioma and breast cancer and correlates with the VEGF expression. MSCs, pericytes and myofibroblasts invade the space between the newly formed membranes of the two vessels and form intervascular tissue pillars or structures that expand the existing vasculature. Vascular remodeling has been shown to be associated with hemodynamics in several animal models, including mouse, rat and zebrafish. Karthik et al. [117] observed in vivo the role of intussusceptive arborization in the zebrafish embryo model, highlighting that this process during shear stress led to complex vascular structures with specific angio-architecture.

In conclusion, the study of all these mechanisms could provide new information on the biology of tumors and could be useful for the development of new anti-tumor therapies aimed at interrupting tumor angiogenesis and tumor plasticity.

## 4. Future Perspectives

VSCs play a crucial role in the resolution of the vascular lesion as well as in neovascularization associated with carcinogenesis, favoring the formation of metastases, survival and the progression of tumors. The upregulation of pro-angiogenic factors and recruitment of VSCs are among the proposed mechanisms for resistance to anti-angiogenic therapy [118]. Consequently, VSCs represent a new target for the treatment of resistance to anti-angiogenic therapy. The identification of VSCs remains an open issue as there is no unambiguous marker available. It is also unclear whether all types of VSCs arise from the same source during development or if they alter in adults, particularly under disease conditions. Further investigations should be explored to understand the molecular, genetic and phenotypic characteristics of these progenitors to define new vascular targeting strategies aimed at personalizing the vessel function by optimizing the drug response in cancer.

## Figures and Tables

**Table 1:** Markers of endothelial progenitor cells in clinical trials.

Marker	Clinical Trials (Analyzed Disease)	Clinical Issue	PMID
cKit+	COVID-19 and bioprosthetic total artificial heart implantation, glioblastoma growth glioblastoma	Atherosclerosis, angiogenesis in cancer, vasculogenesis in cardiovascular diseases	33205351, 24311637, 20821342
CD19+	COVID-19 and bioprosthetic total artificial heart implantation	Vasculogenesis in cardiovascular diseases	33205351
CD31+	Metastatic Renal Cell Carcinoma, ovarian cancer, Hepatocellular Carcinoma, ovarian or peritoneal cancer, epithelial ovarian cancer, metastatic pancreatic cancer, stent implantation	Angiogenesis in cancer, angiogenic sprouting and vessel permeability, tumor microvessel density, microvascularization, metastasis, injury by implantation	34921022, 33103699, 29059426, 26210785, 20870280, 20388201, 20130876, 25915510
CD34+	COVID-19 and bioprosthetic total artificial heart implantation, epithelial ovarian cancer, metastatic pancreatic cancer, hypoxic stress, arterial erectile dysfunction	Vasculogenesis in cardiovascular diseases, metastasis, injury by hypoxia, cavernous arterial insufficiency	33205351, 20388201, 20130876, 27761657, 21868743
CD45+	COVID-19 and bioprosthetic total artificial heart implantation, electronic cigarettes	Vasculogenesis in cardiovascular diseases, increase of endothelial progenitor cells	33205351, 27693003
CD14+	Angiogenic Homeostasis in Diabetes¸ hypoxic stress, electronic cigarettes	Injury in diabetes, injury by hypoxia, increase of endothelial progenitor cells	31102457, 27761657, 27693003
CD105+	Epithelial ovarian cancer, ovarian cancer	Cancer angiogenesis, neoangiogenesis	20388201, 19135712
CD144/VE-cadherin+	Atrial fibrillation, electronic cigarettes¸ oxidative stress and hypoxia by extreme altitude, stent implantation, arterial erectile dysfunction, ingestion of saturated fatty acids	Protrombotic injury, increase of endothelial progenitor cells, injury by hypoxia and oxidative stress, injury by implantation, cavernous arterial insufficiency, injury by saturated fatty acids	33237804, 27693003, 26820158, 25915510, 21868743, 19846183
CD146+	Mediterranean diet with nuts (MedDiet-nuts), coronary artery disease, metastatic colorectal cancer, coronary intervention	Cardiovascular risk, stress-induced arteriogenesis, prognosis, extent of endothelial injury	27052787, 23056467, 21825101, 20846600
CD45-	Metastatic pancreatic cancer, arterial erectile dysfunction	Metastasis formation, cavernous arterial insufficiency	20130876, 21868743
CD146-	Cancer therapy	Prognosis	22028623
Tie2-	Angiogenesis in glioblastoma	Cancer angiogenesis	20821342
vWF+	Hematological malignancy	Thrombosis and bleeding complications.	31390488
VEGFR2+	Angiogenesis in glioblastoma, tumor shrinkage clear-cell metastatic renal cell carcinoma, melanoma, acute myeloid leukemia, advanced cancer	Cancer angiogenesis	20821342, 20215520, 19284623, 19188183, 17169805, 15867205
Flk-1	Metastatic breast cancer, seasonal allergic rhinitis	Tumor progression, angiogenesis in patients with asthma	17651148, 22585426

**Table 2:** Markers of Smooth muscle progenitor cells.

Marker	Clinical Trials (Analyzed Disease)	Clinical Issue	PMID
Gli1+	Metastatic pancreatic cancer, advanced solid tumors, head and neck squamous cell carcinoma	Metastasis formation, prognosis	31787526, 28317088, 21357786
PDGFRβ	Thymic epithelial tumors, meningiomas, advanced renal-cell carcinoma, macular degeneration, malignant meningiomas, metastatic breast cancer, metastatic salivary gland carcinomas	Tumor regression, prognosis, survival, better outcomes, pharmacokinetics, antigiogenic effects	34706060, 31220093, 30374686, 31791663, 31220093, 28464908, 27821319

**Table 3:** Marker of Mesangiogenic progenitor cells.

Marker	Clinical Trials (Analyzed Disease)	Clinical Issue	PMID
Sox2	Myeloma; multiple myeloma; antimyeloma immunity, non-small-cell lung cancer	Tumor progression, tumor prevention (antimyeloma immunity), metastasis formation, prognosis	30830874, 26827660, 23430442, 22837720
Ki-67-	Node-Positive Breast Cancer	Prognosis	31407967
Nestin+	Breast Cancer; myeloproliferative neoplasms; dermatofibrosarcoma protuberans; glioblastoma multiforme	Tumor progression, prognosis, tumor invasion,	34615722, 30409796, 23962157, 20063522

**Table 4:** Markers of perycytes.

Marker	Clinical Trials	Pathological Issue	PMID
CD13+	Diffuse large B-cell lymphoma, primary central nervous system lymphoma, B cell lymphoma, solid tumors	Vascular permeability and CNS access of anticancer drugs, blood-brain barrier (BBB) penetration, diagnostic subtyping, cancer angiogenesis	32766857, 31118164, 30066366, 19900802
α-SMA	Colorectal cancer, nasopharyngeal carcinoma, chronic allograft nephropathy, usual interstitial pneumonia and nonspecific interstitial pneumonia	Metastasis and immune cell activity, neoangiogenesis, allograft injury (transplantation), fibrotic diseases	27248825, 24877105, 16236802, 15955241
NG-2	Neurologic and neurometabolic diseases	Replacement in neurologic diseases	24558163
RGS 5	Metastatic colorectal cancer	Tumor maturation	25069475
Desmin	Calcifying fibrous tumor, esophageal cancer, childhood sarcomas	Diagnosis, tumor progression and depth of invasion, diagnosis	31355265, 23801316, 1710539
DLK-1	Central precocious puberty, lung cancer	Timely diagnosis and treatment, prognostic factor	36577869, 33508526
Endosialin	Metastatic soft-tissue sarcomas, solid tumors, treatment-refractory solid tumors	Tumor angiogenesis, tumor shrinkage,	31034598, 30623276, 25398449

## Data Availability

Not applicable.
